# Vis Medicatrix Naturæ

**Published:** 1894-02

**Authors:** W. H. Whitslar

**Affiliations:** Cleveland, O.


### "Vis Medicatrix Naturæ."

BY W. H. WHITSLAR, M.D., D.D.S., CLEVELAND, O.

Read before the Ohio State Dental Society, December, 1893.

The diseases that the human body is subject to, number into the thousands. The teeth, as important organs of the human economy have diseases peculiar to themselves, also those that are symptomatically observed in remote parts of the body.

Upon first consideration it would appear that there are many diseases of the teeth, but scarcely fifty idiopathic ones can be numbered. This seems, however, to be a great many. Literally, only the organized portions of the teeth may become diseased. The mineral constituents while predominant in quantity are subservient to the organized material interposed. Vital phenomena aid in resisting disease, but external influences may, as in caries, cause disintegration of mineral substances, leaving only the life portions with insufficient material to make a healthy stump.

It is here that the dental surgeon with his sharp excavators and chisels removes the disease.

If perchance there remains enough body to aid retention of foreign materials, like the surgeon who supplies a missing portion of the skull with metallic substances, the dental surgeon inserts fillings for the arrest of decay and restoration of form, the restoration of tooth substance being impossible.

The practice of dentistry is a surgical procedure in the main, since it consists of the manual operations adapted to cure of diseases.

A surgeon aptates tissue, Nature performs the cure.

The dentist cannot aptate the hard tissue because of the quantity being too great to be carried into the circulation or otherwise substantially affected by it. He supplies their loss and the presence of foreign materials is either tolerated or rebuked by the remaining living parts.

Pain is the first rebuke, and efforts to repair are the first fruits of Nature to resist invasion.

The resiliency of Nature is peculiarly strong in the absorption of deciduous teeth, not abstractedly, but for the provision of teeth for mastication in ultimately strong men and women.

We have here growth, growth which is the antitype of repair, and repair which is the repetition of growth. The same elements are necessary to the formation of results in either.

*Rest* is the necessary antecedent to both growth and repair. This principle is noticeable in the vegetable kingdom. "All plants require rest and obtain it in some countries by the rigor of winter, in others by the scorching heat of summer." Said John Hunter, "Some plants close their leaves, others their flowers at particular hours of the day or night, and with such regularity does this period of rest take place that more than one vegetable physiologist has proposed to construct from them, a floral clock."

Repair in the young body is accentuated by conditions of the body and its requirements. It is often necessary in providing remedies for growth and repair, to have a knowledge of the temperament, age, physical condition, occupation and general surrounding circumstances of the individual, as a basis upon which to advise remedial application.

The healing power of Nature has a constant tendency to repair injuries and this is accomplished by interposing material to prevent invasion of further injury or disease.

It is clinically observed that, under certain classes of fillings a hardening of tissue results. This cannot be due to the hardening of each molecule of the mineral substance composing the tooth, but that remoleculization probably is due to vital fluids which may act as the water of crystallization.

In the pulp and tissues about the teeth, Nature provides a coagulable lymph to form a splint, for the purpose of coaptation of divided tissues in order to secure a degree of rest. This inflammatory effusion is not the ultimate bond of union, but holds the parts in apposition, prevents friction, provides against invasion of microbes, and allows nature to dispose of the offending cause. This is why, when we wound the gums as in the removal of tartar, the result of injury to the gums is so frequently *nil.*

The fibrous nature of the gum aids in holding the lypmh, interlocking it in its meshes, thus providing a safeguard against the ravaging microbes of disease, or the toxic properties of the saliva.

There is an inherent power in every cell to recovery, so that, as time elapses, the cells under this seruminal matrix become old enough to withstand outward influences, a solvent then separates the dried matrix and it is cast off. This is precisely what we seek to accomplish in our treatment, i. e., to remove cause of disease and protect the tissues.

It is surprising what Nature will do for us.

Too often the praise is ascribed to the skill of the dentist, though skill is necessary to combat the evils of too much medicament, thus limiting Nature's right to perform its natural functions.

The dental pulp is so sensitive an organ that it is our belief that more pulps die from over medication than from other causes. Rapidity of introduction of various medicaments alternate shocks that at once exhaust the vital energy of the cells also causing congestion of the vessels. It is further our belief that following the death of the pulp, the pulp-canal is made a reservoir of all kinds of fluids supposedly for medication but inviting necrosis of the tissues of the apical space. Abscesses encroach upon the other tissues and destroy them, followed by pain and constitutional disturbances. We open abscesses to relieve these conditions, and evacuate fluid to cause coaptation of the organ zed tissue. Nature will cement them together. Our efforts to cure abscesses are sometimes failures because putrescible substances do not permit coaptation of desirable tissues. In some of these instances our medication though scientifically (?) applied results in dire failure. The object of this paper is not to decry the use of medicines but to express the sentiment that it is a laudable and a progressive profession that is seeking the best remedies for disease.

It is, however, patent that while we favor the correct application of medicaments liberally, let us be conscious of the healing powers of Nature.

---

THE suicide mania has became so great in Denmark, that the government is considering measures to check it. One likely to be adopted is the Swedish law, which compels the body of every person who commits suicide to be sent to the dissecting room of the nearest university.

---

THE Turkish Government prohibits the importation and sale of secret patent medicines within its dominions.

